# Determination of nicotine in tobacco products based on mussel-inspired reduced graphene oxide-supported gold nanoparticles

**DOI:** 10.1038/srep29230

**Published:** 2016-07-04

**Authors:** Yanqiu Jing, Xiuxiu Yuan, Qiu Yuan, Kuanxin He, Yingjie Liu, Ping Lu, Huaiqi Li, Bin Li, Hui Zhan, Guangliang Li

**Affiliations:** 1 College of Tobacco Science, Henan Agricultural University, Zhengzhou, Henan 450000, China; 2 Science and Technology Department of Jiangxi of China National Tobacco Corporation, Nanchang, Jiangxi, 330000, China; 3 Research institute of Jiangxi of China National Tobacco Corporation, Nanchang, Jiangxi, 330000, China; 4 Zhengzhou Branch of Henan Tobacco Corporation, Zhengzhou, Henan, 450000, China; 5 Tobacco Industry Technology Research and Development Center, Zhengzhou, Henan, 450000, China; 6 Quality Management Section of Hainan Hongta Cigarette CO., LTD, Haikou, Hainan, 570100, China

## Abstract

Polydopamine functionalized reduced graphene oxide-gold nanoparticle (PDA-RGO/Au) nanocomposites were successfully prepared by a simple and mild procedure. The PDA-RGO/Au nanocomposite is successfully formed in an aqueous buffer solution (pH 8.5) without using any reducing agent. FTIR confirmed the successful coating of PDA and informed the reduction of the surface functional groups of GO. The formation of reduced GO and Au NPs was further evidenced by UV-Vis and X-ray diffraction spectroscopy. This method is environmentally friendly and highly beneficial for the mass production of graphene-noble metal based nanocomposite. The as prepared PDA-RGO/Au nanocomposite could greatly enhance the electrochemical oxidation of nicotine. We fabricated an electrochemical nicotine sensor based on the prepared PDA-RGO/Au nanocomposite. The proposed nicotine sensor showed a wide detection range from 0.05 to 500 μM with a low detection limit of 0.015 μM. Moreover, the proposed nicotine sensor was also successfully applied for determination nicotine content in tobacco products.

Inspired by the bio-adhesion principle of marine mussels in wet and turbulent environments, Messersmith and his colleagues found that dopamine could spontaneously polymerize under slightly alkaline conditions, leading to the formation of a polydopamine (PDA) coating with secondary reactivity on virtually all substrates^1^. The polymerization of dopamine needs to be initiated by an oxidization reaction^2^,^3^. Lee and co-workers suggested that the formation of PDA was a result of the combination of covalent polymerization and noncovalent self-assembly^4^. During the polymerization process, many functional groups including planar indole units, amino group, carboxylic acid group, catechol or quinone functions, and indolic/catecholic π-systems are integrated into PDA, which provide the robust adhesion capability of PDA. On the other hand, PDA, similar to its monomer, is prone to attach on substrates through noncovalent binding interactions such as metal coordination, hydrogen bonding, π–π stacking, and quinhydrone charge-transfer complexes^5^,^6^. Due to its simplicity and good biocompatibility, PDA coating has recently attracted great interest and was intensively studied for surface modification of nanomaterials. For example, Li and co-workers recently prepared a PDA functionalized reduced graphene oxide-silver nanoparticle and successfully used for H_2_O_2_ sensing^7^. Roy and co-workers prepared a PDA functionalized boron nitride nanosheet-supported gold nanoparticles for catalytic reduction of 4-nitrophenol^8^. It can be seen that the PDA is an excellent candidate for functionalizing other substances. The functionalized materials showed advanced properties in many ways. PDA plays a key role for attempting various types of surface functionalization and for providing several adhesive coatings on a wide range of materials.

Graphene has attracted a great deal of interest since it has been discovered in 2004 due to its extraordinary properties, such as excellent electronic conductivity, large specific surface area and enhanced electrocatalytic activity^9^,^10^,^11^,^12^,^13^,^14^,^15^. Therefore, graphene is considered as an excellent candidate for electrode surface modification for specific target molecule detection. However, most of the graphene used in this procedure is in its reduced form from graphene oxide (GO) prepared by the oxidation of graphite. The reduced graphene oxide (RGO) is prone to irreversible spontaneous agglomeration, which highly limits its applications and performances. To overcome this problem, DA and its derivatives have been studied for reducing and functionalizing GO to a solution-processable RGO^7^,^16^,^17^.

Herein, we report the preparation of PDA-RGO/Au nanocomposite using a one-step mild wet chemical synthesis method. Specifically, a PDA layer was incorporated between GO and AuNP as molecular glue. During the DA polymerization process, the GO and Au^3+^ ions were reduced into RGO and Au NPs, respectively, without the use of a reducing agent. The entire method for PDA-RGO/Au nanocomposite preparation can be proceeded in an aqueous media without the use of any additional chemical reagents, which is favourable for green scale-up and mass-production. The morphology and structure of the prepared materials are characterized by FTIR, UV-vis spectroscopy, SEM, XPS and XRD. The prepared PDA-RGO/Au nanocomposite was successfully used for electrode surface modification and then applied for electrochemical determination of nicotine in the tobacco products.

## Experiments

### Chemicals and materials

3-hydroxytyramine hydrochloride (DA) and HAuCl_4_ were purchased from Sigma-Aldrich. Graphene oxide powder was purchased from JCNANO, INC. Nicotine standard samples (99%) were provided by the Chinese tobacco industrial company of Henan. Britton–Robinson (BR) (0.1 M) supporting electrolyte buffer solutions of pH range (2.0–8.0) (CH_3_COOH+H_3_BO_3_+H_3_PO_4_) were used for preparing the standard solutions of nicotine. Other chemicals were of analytical reagent grade and used without further purification.

### Synthesis of PDA-RGO/Au nanocomposite

For synthesis of PDA-RGO/Au composite, 10 mg GO and 0.5 mL of aqueous solution of HAuCl_4_·3H_2_O (50 mM) were dispersed in 30 mL Tris-buffer (10 mM, pH 8.5) and sonicated for 1 h. 10 mg DA was then added into the dispersion and sonicated for another 10 min. The resulting mixture was kept stirring for 24 h. The PDA-RGO/Au product was collected and washed three times by water using centrifugation. PDA functionalized RGO (PDA-RGO) was synthesized using a similar method without adding HAuCl_4_·3H_2_O.

### Characterization

The morphology and structure of the prepared samples were characterized using a field emission scanning electron microscope (FeSEM, ZEISS SUPRA 40VP, Germany) and an X-ray diffractometer (D8 –Advance XRD, Bruker, Germany) with Cu Kα radiation, respectively. UV-vis spectra of samples were collected by UV-Vis spectroscopy (HALO RB-10, Dynamica) in the wavelength range from 200 to 700 nm. Fourier transform infrared spectroscopy (FTIR, Nicolet iS5, Thermo Scientific, USA) was used for analysing surface functional groups of the sample. X-Ray photoelectron spectroscopy (XPS) spectra were recorded with a PHI Quantera II Scanning XPS Microprobe (Physical Electronics Inc).

### Electrode modification

For glassy carbon electrode (3 mm in diameter) modification, 3 μL of PDA-RGO or PDA-RGO/Au nanocomposite dispersion (1 mg/mL) was dropped onto the GCE surface and dried at room temperature. Then, 7 μL of Nafion (1 wt% in ethanol) was placed onto the GCE surface to obtain the PDA-RGO/Au modified GCE. Electrochemical measurements were performed on a CHI430A electrochemical workstation (USA) using a three electrode system was used for the experiment. An Ag/AgCl (3 M KCl) as the reference electrode and a platinum electrode was used as the auxiliary electrode.

### Cigarette sample preparation

Commercial cigarettes were purchased in a local cigarettes shop. The cigarettes were freed from the rolling paper and the filter. Tobacco from ten cigarettes of each brand was mixed and dried in an oven. 1 g tobacco powder was put into a 50 mL beaker, then 20 mL of deionized water was added and the receptacle was capped. The mixture was sonicated for half hour in an ultrasonic water bath at room temperature, and then the slurry was filtered. The clear filtrate was collected as sample^18^.

## Results and Discussion

### Characterization of PDA-RGO/Au nanocomposite

**Figure 1:**
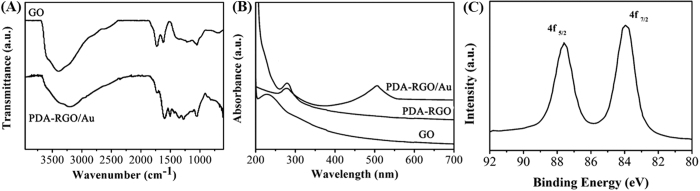
(**A**) FTIR spectra of GO and PDA-RGO. (**B**) UV-vis spectra of GO, PDA-RGO and PDA-RGO/Au nanocomposite. (**C**) XPS Au 4f narrow scan of PDA-RGO/Au nanocomposite.

It has been reported that dopamine can undergo self-polymerization to form adherent PDA in a weak basic condition. Herein, we use this method to functionalization of GO and reduction of Au salt with PDA. The reduction of GO using PDA was characterized by FTIR spectroscopy. The IR spectrum (Fig. 1A) of GO before coating presents peaks at 1732, 1622, 1395 and 1049 cm^−1^, which are assigned to the C=O stretching of COOH groups, C=O stretching vibration, C-OH stretching vibration and C-O vibrations from alkoxy groups, respectively. After coating with PDA, the intensity of these peaks becomes much less, indicating that the amount of oxygen-containing groups at the surface of GO is greatly reduced. Dopamine and its derivatives have been used as reducing agents, it is possible that the oxygen groups at the surface of GO are chemically reduced by dopamine during the polymerization process^19^. Furthermore, two new peaks featured at 1507 and 1351 cm^−1^ are observed for PDA-RGO sample. These two peaks are due to the stretching vibration of C=N and C-N-C of indole ring, which confirm the successful modification of PDA on GO sheets^20^. The reduction of GO using PDA was also confirmed by the UV-vis spectroscopy. Figure 1B shows the UV-Vis spectra of water dispersion of GO, PDA-RGO and PDA-RGO/Au nanocomposite. The GO spectrum exhibits a characteristic absorption peak at 229 nm corresponding to the π → π* transition of aromatic C=C bonds. After functionalization with PDA, this peak shifts from 229 to 282 nm, giving further evidence that most GO has been reduced to RGO^21^. Though the absorbance of the whole visible range has a big increase, the dispersion of PDA-RGO does not show any perceptible precipitation, implying that the formed PDA is a great stabilizer to prevent the stacking of the reduced graphene sheets. The formation of Au NPs was also confirmed by the UV-vis spectra analysis. The spectrum of PDA-RGO/Au nanocomposite shows a clear broad absorption peak centred at 510 nm, which is due to the surface plasmon resonance absorption of Au NPs, indicating the simultaneous formation of Au NPs. The formation of Au NPs was further confirmed by the XPS analysis. As shown in the Fig. 1C, the deconvoluting of the Au4f spectrum showed two overlapping binding energies of 82.1 eV (Au4f_7/2_) and 85.6 eV (Au4f_5/2_), which confirms the existence of metallic Au^0^ in PDA-RGO/Au nanocomposite.

**Figure 2:**
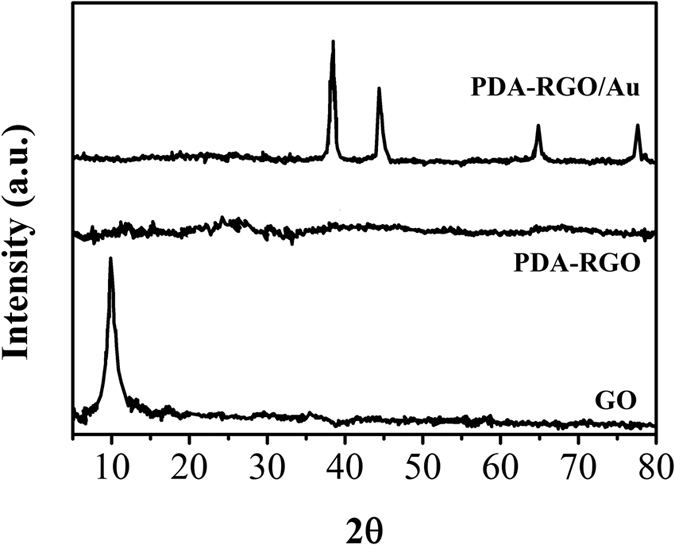
XRD pattern of the GO, RGO and PDA-RGO/Au nanocomposite.

The interlayer changes and crystalline structure of GO, RGO and PDDA-RGO/Au nanocomposite were analyzed by XRD and depicted in Fig. 2. The GO exhibits a typical characteristic (001) peak at 11.1°. This peak is not seen for PDA-RGO/Au nanocomposite, indicating the GO has been reduced after the functionalization with PDA. A small peak centred at 22.8° is due to the presence of stacked graphene layers of RGO^22^. The XRD spectrum of In PDA-RGO/Au nanocomposite shows diffraction peaks located at 39.7°, 460°, 67.2° and 81.3°, which are assigned to (111), (200), (220) and (311) planes of face-centered-cubic (fcc) crystallographic structure of Au (JCPDS 4–0783), respectively, confirming the successful electrodeposition of Au nanoparticles.

**Figure 3:**
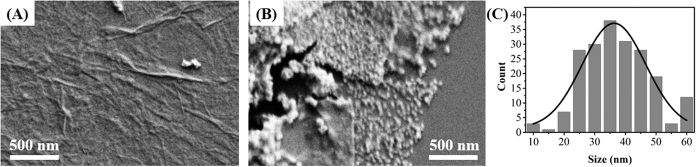
SEM images of (**A**) GO and (**B**) PDA-RGO/Au nanocomposite. (**C**) Particle size distribution of Au NPs.

In order to examine the morphology of the PDA-RGO/Au nanocomposite, SEM analysis has been conducted and the images are shown in Fig. 3. As shown in the Fig. 3A, the GO displayed a thin layer with winkle morphology. In comparison with the pristine GO sheets, the PDA-RGO/Au nanocomposite (Fig. 3B) shows uniform decoration of Au nanoparticles on both sides of the PDA-RGO sheets. The average particle size of Au is about 35.7 nm (based on 200 individual Au NPs and the Gaussian function was used to fit the distribution) (Fig. 3C).

### Electrochemical determination of nicotine

**Figure 4:**
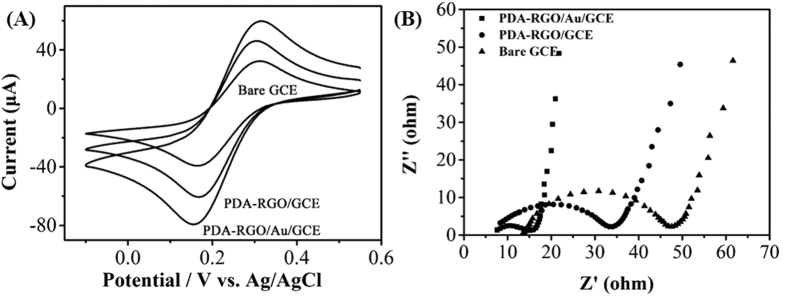
(**A**) Cyclic voltammograms of bare GCE, PDA-RGO/GCE, PDA-RGO/Au/GCE in 20 mM Fe(CN)_6_^3−/4−^ with 0.2 M KCl. (**B**) Nyquist diagrams of bare GCE, PDA-RGO/GCE and PDA-RGO/Au/GCE in 2 mM K_4_[Fe(CN)_6_] + 0.2 M KCl.

Cyclic voltammetry (CV) and electrochemical impedance spectroscopy (EIS) were used for analysing the electrochemical properties of the bare GCE, PDA-RGO/GCE and PDA-RGO/Au/GCE. Figure 4A shows the cyclic voltammograms (CVs) of bare GCE, PDA-RGO/GCE and PDA-RGO/Au/GCE using Fe(CN)_6_^3−/4−^ redox probe. A pair of well-defined redox peaks could be observed during the all CV scans. Compared with the bare GCE, the peak intensities of the PDA-RGO/GCE were slightly increased, suggesting that the electron conductivity of PDA-RGO modified GCE is better than that of the bare GCE, and thus that the PDA-RGO modification enhanced the electron transfer between the electrode and solution to some extent. In contrast, the PDA-RGO/Au/GCE showed a much higher current responses to the probes compared with other electrodes, suggesting the enhanced electroactive surface area as a result of the high electron-conductivity. The electroactive surface area of the electrode can be calculated according to the Randles-Sevcik equation:


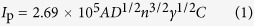


where *A, D, n, γ and C* is the area of the electrode (cm^2^), diffusion coefficient of the molecule (cm^2^/s), number of electrons transfer in the redox reaction, scan rate (V/s) and concentration of the probe molecule (mol/cm^3^), respectively. In Fe(CN)_6_^3−/4−^ redox system, the *D* is equal to 6.7 × 10^−6^, the *n* is equal to 1, the *γ* is equal to 0.02 and the *C* is equal to 20. The electroactive surface of bare GCE, PDA-RGO/GCE and PDA-RGO/Au/GCE was calculated as 1.4 × 10^−3^, 6.25 × 10^−3^, and 2.33 × 10^−2^ cm^2^, respectively. Therefore, the surface area of PDA-RGO/Au/GCE is 16.6 times larger than that of the bare GCE.

Figure 4B shows the Nyquist plots of the bare GCE, PDA-RGO/GCE and PDA-RGO/Au/GCE. EIS is an efficient tool for studying the interface properties of surface-modified electrode. The electron-transfer resistance (R_ct_) at the electrode surface is equal to the semicircle diameter of EIS, and can be used to describe the interface properties of the electrode. As shown in the figure, a big semicircle of about 48 Ω diameter with an almost straight tail line are present at the bare GCE, demonstrating very low-electron-transfer resistance to the redox-probe dissolved in the electrolyte solution. It also can be seen that a smaller semicircle diameter was observed in the PDA-RGO/GCE, suggesting the enhanced electroactivity. Interestingly, the R_ct_ from PDA-RGO/Au/GCE markedly decreased to about 14 Ω, which should be due to the fact that the incorporation of Au NPs largely enhanced the conductivity of the electrode. The obtained R_ct_ values have confirmed the electroactivity sequence of the results obtained from the CV experiments. Based on the above characterizations, the Au NPs are distributed within PDA-RGO film as tiny conduction centers, which can accelerate the electron transfer between the probe molecules and GCE.

**Figure 5:**
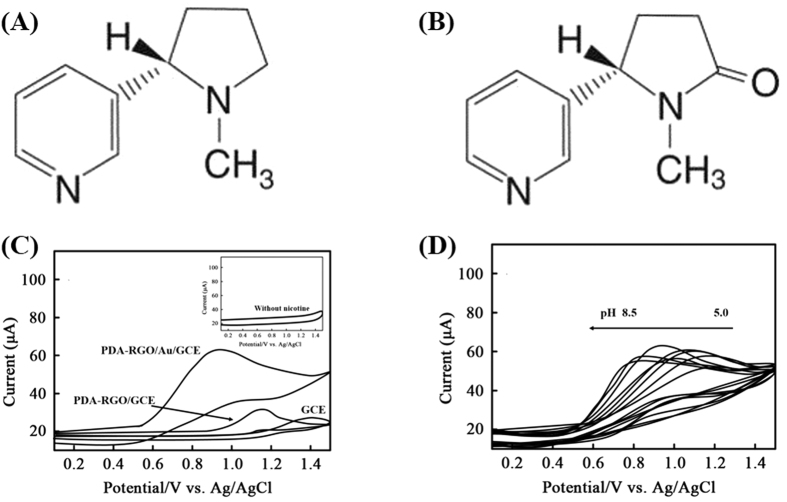
Chemical structure of (**A**) nicotine and (**B**) cotinine. (**C**) Cyclic voltammograms of bare GCE, PDA-RGO/GCE and PDA-RGO/Au/GCE toward 50 μM nicotine in BR (pH = 7.5) with scan rate of 50 mV/s. Inset: PDA-RGO/Au/GCE scan in BR without nicotine. (**D**) Cyclic voltammograms of 50 μM nicotine at PDA-RGO/Au/GCE in BR pH 5.0–8.5 at scan rate of 50 mV/s.

As the primary neuroactive alkaloid in tobacco, nicotine (Fig. 5A) has been the focus of a great deal of research in recent decades, and has been found to have an increasing number of harmful effects on human health^23^,^24^. The electroanalytical detection of nicotine or its primary metabolite, cotinine (Fig. 5B), is not straightforward. Nicotine appears to display irreversible electrode kinetics on most electrode surfaces, with any oxidative features obscured by solvent breakdown or surface oxidation. Therefore, we attempted to detecting nicotine based on PDA-RGO/Au nanocomposite due to its outstanding electrochemical properties.

The following experiments concerned whether a PDA-RGO/Au/GCE could be applied for quantitative detection of nicotine. Figure 5C shows the CVs of bare, PDA-RGO/GCE and PDA-RGO/Au/GCE in pH 7.5 BR with absence and presence of 50 μM nicotine. A small oxidation peak was observed in the GCE with potential of 1.2 V. In contrast, PDA-RGO/GCE showed a small peak potential shift from 1.4 V to 1.2 V, suggesting the electrode surface modification using PDA-RGO enhanced the conductivity of the electrode. Moreover, the presence RGO may also attributed electrocatalytic oxidation reaction of the nicotine. A much significant current enhancement with further oxidation potential shift was observed at the CV scan using PDA-RGO/Au/GCE. As shown in the figure, the PDA-RGO/Au/GCE showed a well-defined oxidation peak at 0.93 V with current value of 66.5 μA. The incorporation of uniform Au NPs into the PDA-RGO sheets highly enhanced the electrocatalytic property of the electrode towards nicotine oxidation.

CVs were collected in the presence of 50 μM nicotine in BR of pH range 5.0–8.5 to check the effect of pH on the electrochemical behaviour of PDA-RGO/Au/GCE. As expected, the nicotine oxidation process is pH dependant. Figure 5D shows the CVs of PDA-RGO/Au/GCE towards 50 μM nicotine with different pH BR. The oxidation peak current increased with increasing pH of the BR until a maximum value was achieved at pH 7.5. Moreover, the oxidation peak potential shifted to a negative direction (lower positive potential) with increasing pH, suggesting protons are involved in the electrode process. Peak splitting was observed at several electrode modifiers for nicotine detection^25^,^26^. No peak splitting was observed during the pH change in our case, which is in agreement with the reports for the oxidation of nicotine at molecularly imprinted TiO_2_-modified electrode^27^, boron-doped diamond electrode^28^ and multi-walled carbon nanotube–alumina-coated silica nanocomposite modified electrode^29^. This phenomenon indicates the electrode itself did not undergo electrochemical reaction under the scan potential, which is favourable for electrochemical determination by elimination of disturb signals.

**Figure 6:**
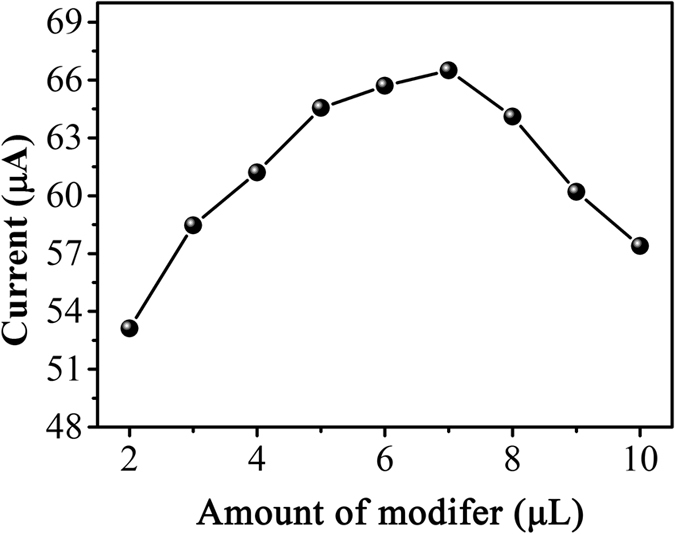
Effect of solution pH on the oxidation potential and peak current of 50 μM nicotine at the PDA-RGO/Au/GCE.

The influence of the amount of the electrode modifier in electrochemical detection performance was studied. Figure 6 shows the effect of different amount of PDA-RGO/Au nanocomposite on the oxidation peak current of nicotine. It can be seen that the oxidation peak current increased with increasing amount of modifier until a maximum value was achieved at 7 μL. Further increasing the PDA-RGO/Au nanocomposite resulted a decreasing of oxidation current due to the thicker layer of modifier hinder the electron transfer rate. Therefore, 7 μL of PDA-RGO/Au nanocomposite dispersion was used for GCE surface modification.

**Figure 7:**
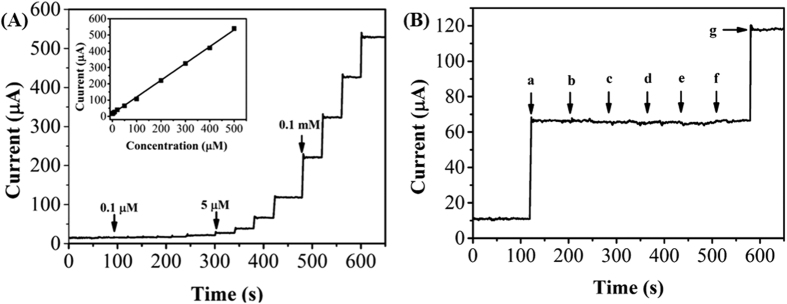
(**A**) Amperometric response of the PDA-RGO/Au/GCE with successive additions of nicotine. Measured at 0.93 V. Inset: Plots of nicotine concentration and current response. (**B**) Amperometric current response of PDA-RGO/Au/GCE to the addition of 50 μM nicotine (a) 1 mM cotinine (b) 1 mM ascorbic acid (c) 1 mM uric acid (d) 1 mM dopamine (e) 1 mM H_2_O_2_ (f) and 50 μM nicotine (g) at an operating potential of 0.93 V.

Figure 7A shows the typical amperometric response upon successive additions of nicotine at PDA-RGO/Au/GCE. It can be seen that the PDA-RGO/Au/GCE attains a steady state current within in 7 s when the potential fixed at 0.93 V, suggesting the fabricated sensor has a rapid response towards nicotine. A linear relationship between the current response and the UA concentrations was observed in the range of 0.05 and 500 μM. The linear regression equation can be represented as: *I* (μA) = 1.032 C(μM) + 15.573 (R^2^ = 0.998). The detection limit was calculated to be 0.015 μM (S/N = 3). The comparative study (Table 1) indicates that the proposed sensor could be applied for detecting the nicotine content sensitively. The high electrocatalytic activity of the prepared nanocomposite towards nicotine detection can be described in several aspects. Firstly, Au nanoparticle itself had an outstanding electrocatalytic property towards many electro-active molecules. However, pure Au nanoparticles always exhibit aggregation without any surfactant and lose their surface area. The introduction of surfactant may keep the high surface area of the Au nanoparticle but decrease the electrocatalytic property at the same time. The *in situ* growth of Au nanoparticles on PDA functionalized RGO could effectively solve the aggregation problem of the Au nanoparticles. Then, the PDA functionalized RGO could prevent the re-stacking effect of the RGO and provide an excellent platform for large number of Au nanoparticles immobilization. Based on the merits of the each component, the fabricated PDA-RGO/Au/GCE shows a sensitive electrocatalytic activity towards nicotine determination.

In the human body, cotinine has been reported to be the principal metabolite of nicotine in blood and urine. In order to investigate the selectivity of the proposed sensor, the influence of cotinine and some physiological interferents has been studied. Figure 7B shows the typical amperometric response of PDA-RGO/Au/GCE upon the addition of nicotine, and a series of potential interference species including cotinine, ascorbic acid, uric acid, dopamine and H_2_O_2_. No obviously change in the current response after addition of 1 mM cotinine, ascorbic acid, uric acid, dopamine and H_2_O_2_, indicating that the proposed sensor is highly selective towards the determination of nicotine even in the presence of 20-fold excess of common interference species.

The reproducibility of the proposed nicotine sensor was tested by detection of 50 μM nicotine using ten PDA-RGO/Au/GCEs. The relative standard deviation (RSD) obtained was 1.57%, indicating the excellent reproducibility of the proposed nicotine electrochemical sensor. The stability test of the proposed nicotine sensor was tested by 10000 s continuously I-T test in 50 μM nicotine. The responses showed that the proposed PDA-RGO/Au/GCE decreased about 6.3% current response. To evaluate the long-term storage stability, PDA-RGO/Au/GCE was tested by storing in fridge for four weeks. The current responses showed the PDA-RGO/Au/GCE still remains 92.8% of their original activity. Therefore, the proposed nicotine sensor has a satisfactory stability and reproducibility.

The real application of fabricated PDA-RGO/Au/GCE was investigated by determining nicotine content in the tobacco products. Two brands of cigarette and one cigar were chosen for testing the validation of the proposed electrochemical sensor. Standard addition method was applied for real sample test. Table 2 shows the detection results of proposed PDA-RGO/Au/GCE compared with a reference method (RP-HPLC)^30^. It can be seen that the proposed PDA-RGO/Au/GCE exhibited an outstanding electrochemical sensing towards nicotine detection in real commercial tobacco products, indicating the proposed PDA-RGO/Au/GCE could be potentially used for detecting nicotine content in the real samples.

## Conclusion

In this contribution, we have demonstrated a simple and mild procedure for preparing PDA-RGO/Au nanocomposite. A series of techniques including UV-vis spectroscopy, FTIR, SEM and XPS were used for characterizing the prepared PDA-RGO/Au nanocomposite. CV and EIS characterization results found the prepared PDA-RGO/Au nanocomposite owing an excellent electrocatalytic activity towards determination of nicotine. Thus, a sensitive, selective and reliable electrochemical nicotine sensor was fabricated by surface modification of GCE using PDA-RGO/Au nanocomposite. The proposed nicotine sensor exhibited a linear response range from 0.05 to 500 μM and a low detection limit of 0.015 μM. Moreover, the proposed nicotine sensor successfully applied for detecting nicotine content in the tobacco product.

## Additional Information

**How to cite this article**: Jing, Y. *et al.* Determination of nicotine in tobacco products based on mussel-inspired reduced graphene oxide-supported gold nanoparticles. *Sci. Rep.* **6**, 29230; doi: 10.1038/srep29230 (2016).

## Figures and Tables

**Table 1:** Comparison of the PDA-RGO/Au/GCE electrochemical sensor and reported nicotine sensors.

Electrode	Linear range (μM)	LOD (μM)	Reference
MWCNT	31–220	7.6	31
Carbon paste	50–500	6.1	32
Boron-doped diamond electrode	9.9–170	6.1	33
Pencil graphite electrode	7–107.5	2	26
Electrochemically activated GCE	1–200	0.7	34
Molecularly imprinted TiO_2_-modified electrodes	0–5000	4.9	27
CuNPs	1–90	0.164	35
Poly(4-Amino-3-Hydroxynaphthalene Sulfonic Acid)	1–200	0.866	25
MWCNT–alumina-coated silica	5–400	1.42	29
PDA-RGO/Au	0.05–500	0.015	This work

**Table 2:** Determination of nicotine content in two brands of cigarettes and pharmaceuticals using PDDA-RGO/Au/GCE.

Sample	Addition (μM)	Found (μM)	RSD (%)	Recovery (%)	RP-HPLC	RSD (%)
Cigarette 1	0	10.11	3.14	—	10.04	3.22
	20	30.17	4.29	100.20	29.54	1.05
	50	61.01	0.89	101.50	60.15	0.68
	100	112.08	2.21	101.79	110.66	1.43
Cigarette 2	0	5.08	2.33	—	4.92	1.95
	5	10.25	1.59	101.69	9.93	2.14
	10	14.89	1.50	98.74	14.87	2.99
	30	34.91	1.72	99.52	35.12	2.25
Cigar	0	20.09	1.93	—	20.14	3.61
	20	40.12	2.85	100.07	40.16	2.44
	50	70.08	3.70	99.99	70.05	5.21
	100	119.77	3.28	99.73	121.41	3.64
